# The IMIRA project – Improving Health Monitoring in Migrant Populations

**DOI:** 10.17886/RKI-GBE-2018-051

**Published:** 2018-05-16

**Authors:** Susanne Bartig, Alexander Rommel, Claudia Santos-Hövener, Patrick Schmich, Ursula von Schenck, Antje Gößwald, Thomas Lampert

**Affiliations:** Robert Koch Institute, Berlin, Department of Epidemiology and Health Monitoring

A population-specific approach to health is essential if health-policy measures are to reflect the needs of particular target groups. However, in the case of people with a migration background, health reporting faces a major challenge: availability of reliable data. The project ‘Improving Health Monitoring in Migrant Populations’ (IMIRA), which is currently being carried out at the Robert Koch Institute (RKI), aims to improve the information base regarding migrants’ health in Germany (duration: July 2016 – June 2019). In addition to the improvement of inclusion of migrant populations into health monitoring, IMIRA also aims to integrate migrants’ health into regular health reporting.

In order to achieve these goals, several subprojects are being implemented in parallel. An initial review of the current research findings in the field of migration and health has already been undertaken. A further subproject aims to review and assess the applicability of migration-specific concepts (such as acculturation) to health monitoring at the RKI. Two feasibility studies are also carried out to improve the inclusion of migrants into health monitoring. Whereas the feasibility study ‘interview survey’ focuses on testing different recruitment strategies to increase the participation of migrants (such as by providing a multilingual study hotline, and field follow-ups for non-response), the feasibility study ‘examination survey’ includes various tools (such as live video interpreters, multilingual videos describing the examinations) aimed at overcoming language and cultural barriers between participants and the medical staff. The insights gained from the IMIRA project will be taken into account when designing future health surveys at the RKI.

To develop a concept outlining how to integrate data on migrants’ health into regular health reporting is also part of IMIRA. The aim is still to develop a core indicator system that describes the health of migrants. At the same time, and in addition to the data from the health monitoring conducted at the RKI, routine data (such as data from statutory health insurance funds ) and survey data (such as from the Socio-Economic Panel) are to be verified with regard to their usability for migration-related health reporting.

Moreover, networking with relevant actors in the field of migration and health is an ongoing project activity.

